# Monitoring the effect of belinostat in solid tumors by H4 acetylation

**DOI:** 10.1111/j.1600-0463.2008.00957.x

**Published:** 2008-05

**Authors:** LENA MARQUARD, KAMILLE DUMONG PETERSEN, MORTEN PERSSON, KIRSTEN DAMGAARD HOFF, PETER BUHL JENSEN, MAXWELL SEHESTED

**Affiliations:** 1 Experimental Pathology Unit, Copenhagen University HospitalCopenhagen, Denmark; 2 Topotarget A/S, Copenhagen University HospitalCopenhagen, Denmark; 3 Dako A/S, Glostrup, Copenhagen University HospitalCopenhagen, Denmark; 4 Department of Oncology, Copenhagen University HospitalCopenhagen, Denmark

**Keywords:** Histone deacetylase inhibitor, monitoring, histone acetylation, immunohistochemistry, fine needle biopsy

## Abstract

Histone deacetylase (HDAC) inhibition is a novel entity in medical oncology, and several HDAC inhibitors are in clinical trials. One of them is the hydroxamic acid belinostat (PXD101) that has demonstrated therapeutic efficacy for several clinical indications. Acetylation of histones is a key event after treatment with HDAC inhibitors, and could thus be used as a marker for monitoring cellular response to HDAC inhibitor treatment. Here we describe the utility of a newly described monoclonal antibody against acetylated H4 for immunohistochemistry on paraffin-embedded fine needle biopsies from nude mice carrying A2780 human ovarian cancer xenografts. Acetylated H4 was monitored *in vivo* by immunohistochemistry during treatment with belinostat, and compared with pharmacokinetics in plasma and tumor tissue. We found an increased level of acetylated H4 15 min after a single treatment (200 mg/kg i.v.) with maximum level reached after 1 h. H4 acetylation intensity reflected the belinostat concentration in plasma and tumor tissue. The threshold level for belinostat activity, indicated by acetylated H4, correlated with belinostat plasma concentrations above 1,000 ng/ml. In conclusion, examination of H4 acetylation in fine needle biopsies using the T25 antibody may prove useful in monitoring HDAC inhibitor efficacy in clinical trials involving humans with solid tumors.

Histone deacetylase (HDAC) inhibitors are a promising group of anticancer agents that target the group of HDAC enzymes reported to be involved in cancer development (1–3). HDACs lead to transcriptional repression through alteration of chromatin structure. Remodeling of chromatin through modifications of histone molecules is a key event in gene transcription (4). Histones can be modified by acetylation, methylation, phosphorylation, and ubiquitination (4, 5). Acetylation of histones is regulated by the opposite mechanisms of HDACs and histone acetyltransferases (HATs). In general, histone acetylation through HAT activity leads to increased transcriptional activity, whereas deacetylation through HDAC activity leads to transcriptional repression (6, 7).

Treatment with HDAC inhibitors represses HDAC activity and upregulates histone acetylation leading to transcription of genes involved in cell cycle arrest, differentiation, and apoptosis (8–11). Several HDAC inhibitors have proven efficacy in clinical trials (12–18) and in October 2006 the Food and Drug Administration (FDA) approved the first HDAC inhibitor ZOLINZA™ (vorinostat, suberoylanilide hydroxamic acid, SAHA) for treatment of patients with cutaneous T-cell lymphomas (http://www.fda.gov/bbs/topics/NEWS/2006/NEW01484.html).

Histone acetylation is a defining event after treatment with an HDAC inhibitor, and can thus be used as an indicator of HDAC inhibitor activity in both normal and tumor cells. This has led to the widespread use of histone acetylation in peripheral blood mononuclear cells (PBMCs) as a surrogate marker in phase I clinical trials (14, 18–21). However, it would obviously be an advantage to be able to monitor acetylation in a solid tumor and correlate the efficacy of the HDAC inhibitor with concentrations found in plasma. This would provide a valuable tool for investigating alternative schedules and for optimizing the efficacy of HDAC inhibitors in the treatment of solid tumors.

In order to obtain a validated model for this approach, we describe the utility of using a new monoclonal antibody to H4 acetylation for immunohistochemistry, together with its applicability in repeated fine needle biopsies from mouse xenograft solid tumors treated intravenously with the HDAC inhibitor belinostat (PXD101). We further investigated to what extent the time course of H4 acetylation in tumor tissue correlated with the belinostat concentration in plasma.

## MATERIALS AND METHODS

### Cell lines

The following cell lines were purchased from ATCC: HCT-116 human colon carcinoma (ATCC, CCI-247), MCF-7 human breast carcinoma (ATCC, HTB-22) and A549 human lung carcinoma (ATCC, CCL-185). PC-3 human prostate cancer cell line (ECACC, 90112714) was purchased from ECACC. A2780 human ovarian carcinoma cell line was a gift from R. Ozols, Fox Chase Cancer Center, Philadelphia, PA, and P388 murine leukemia cell line was a gift from F.M. Shabel, Southern Research Institute, Birmingham, AL.

### Test of antibody specificity and reactivity

We used the novel mouse monoclonal anti-human T25 antibody against acetylated histone H4 that recognizes acetylated Lys8 and Lys12 on H4 (22). Western blotting using either whole cell lysates of HCT-116 cells or histones purified from P388 cells after treatment with 1 μM belinostat for 1 h was used for testing antibody specificity. Blots were incubated with T25 antibody before development. The appearance of only one band verified antibody specificity. The reactivity of T25 was evaluated according to DAKO standard IHC-procedures on different malignant tumors (head and neck, cervix, brain, colon, stomach, prostate, lymphomas, melanomas, lung, ovary, liver, and breast) as well as on normal tissue (prostate, kidney, pancreas, cerebellum, and tonsil).

### Xenografts

Female NMRI nude mice (Taconic), 6–8 weeks of age, were used for the xenograft models. Tumor cells grown in cell cultures were trypsinized and ∼10^7^ cells (100 μl) were mixed with 100 μl matrigel. Mice were anesthesized using Hypnorm/Dormicum, and cells were injected subcutaneously into the flank.

### Immunohistochemistry

**Fig. 1:**
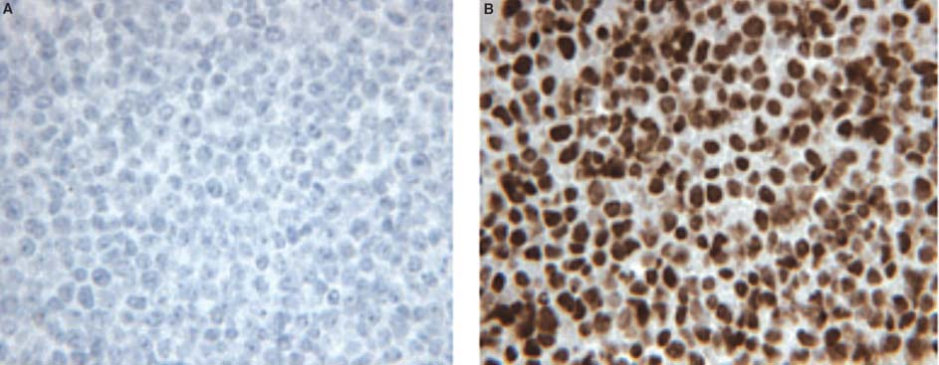
Paraffin-embedded blocks of A2780 ovarian cancer cells were used as controls in immunohistochemical analysis of acetylated H4. (A) Untreated A2780 cells show no H4 acetylation and are used as negative control. (B) A2780 cells treated with 1 μM belinostat for 1 h show strong H4 acetylation and are used as positive control.

Formalin-fixed and paraffin-embedded tumors and biopsies were stained for H&E, p21 and acetylated H4 by standard procedures. For examination of H4 acetylation and p21 expression, heat-induced epitope retrieval was performed using TEG buffer pH 9 (10 mM Tris+0.5 mM EGTA). Peroxidase Blocking Reagent (DAKO, S2001) was used for blocking endogen peroxidase activity. Slides were pre-incubated in 2% BSA in TBS pH 7.6, before primary antibody was added. Primary antibodies were monoclonal anti-p21 (ab16767 [EA10], Abcam, dilution 1:80) and monoclonal anti-acetylated H4 (T25, Topotarget A/S, dilution 1:13,000). The antibodies were diluted in 2% BSA in TBS pH 7.6. EnVision+ (DAKO, K4001) and DAB+ (DAKO, K3468) were used as detection system. Slides were counterstained with Mayer's hematoxylin. Paraffin-embedded blocks of A2780 cells, either untreated or treated with 1 μM belinostat for 1 h, were used as negative and positive control, respectively (see Fig. 1). Slides were scored for staining intensity, i.e. negative (score=0), weak (score=1), moderate (score=2), or strong (score=3).

### Extraction and analysis of belinostat in plasma and tissue

Extraction procedures for plasma samples were conducted to separate any tissue/protein from the sample before analysis. The extractions were carried out using Waters SiroccoTM Protein Precipitation Plates® with 96-well collection plates. First, 150 μl internal standard (13-C-Belinostat) and 50 μl plasma sample was added to each well and shaken on a vortexer. The loaded plate was centrifuged for 5 min, 2000 rpm. All liquid was evaporated under heated nitrogen and the samples were re-dissolved in 100 μl 10% HCOOH in acetonitrile (ACN) (starting eluent) before analysis.

Tumor and spleen samples were homogenized in 400 μl homogenization buffer (25:25:25 Water/ACN/Methanol with internal standard, 13-C-Belinostat 1 μg/ml) using an Ultra Turax® mechanical homogenizer. Samples were then centrifuged at 5000 rpm for 5 min and 150 μl supernatant was removed and kept for analysis.

Quantitative analysis of plasma, tumor and spleen samples was done by liquid chromatography tandem mass spectrometry using Waters UPLC® with reversed-phase analytical column (Acquity C18, 2.1×50 mm, 1.7 μm particle size). A 5 min gradient using 0.05% HCOOH and ACN as mobile phase A and B, respectively, was used with a flow of 0.5 ml/min. All data were processed using MassLynx 4.1.

### Experiment 1: Monitoring the effect of HDAC inhibitor treatment *in vivo* in different xenograft models

The effect of belinostat *in vivo* was tested in a number of different xenograft models (PC-3, HCT-116, MCF-7, A549 and A2780) in order to select a model for biopsy sampling. Mice were treated with 100 mg/kg belinostat or vehicle control (L-arginine 200 mg/kg in isotonic sterile saline). After 1 h, mice were sacrificed and tumors were excised and prepared for immunohistochemistry.

### Experiment 2: Collection of tumor biopsies and influence on H4 acetylation

Tumor biopsies were collected by insertion of an 18G needle into the tumor tissue and carefully aspirating while rotating the needle. It was investigated whether the biopsies taken were representative for the whole tumor and whether the method for biopsy collection had any influence on H4 acetylation. Mice with HCT-116, A2780, PC-3, or MCF-7 tumors were treated with belinostat, 100 mg/kg, and after 1 h they were sacrificed, tumor biopsies were collected, and biopsies and remaining tumor tissue were prepared for immunohistochemistry. For the A2780 model, the effect of repeated biopsy sampling was also investigated. The mice were anesthetized by isoflurane inhalation while tumor biopsies were collected.

### Experiment 3: Time dependence of belinostat treatment on H4 acetylation in solid tumor

The A2780 tumor model exhibited a limited amount of necrosis, and this model was thus selected for further investigation of the relationship between exposure time and H4 acetylation in tumor tissue. Furthermore, the expression of p21 was examined to investigate a possible correlation between H4 acetylation and activation of gene transcription. 16 mice with either small (300–400 mg) or large (1500–1800 mg) A2780 tumors were treated with 100 mg/kg belinostat i.v. at time zero. One pretreatment (only large tumor) and one post-treatment biopsy were collected from the mice. Biopsies were collected at different time points from 1 to 6 h after treatment, as described in Table 1. At sacrifice (3, 6, or 24 h) the entire tumor was removed. Sets of biopsies and corresponding tumors were prepared for immunohistochemistry.

**TABLE 1:** Collection of biopsies for monitoring the effect of belinostat treatment (Experiment 3)

	Large tumors (1500–1800 mg)		
A2780ID	Pretreatment biopsy	PXD101 exposure(h) biopsy	Whole tumor(h)
235	X	1	3
234	X	1	3
226	X	2	3
365	X	2	3
232	X	3	3
233	X	6	6
553	X	6	6
	Small tumors (300–400 mg)		
A2780ID	Pretreatment biopsy	PXD101 exposure(h) biopsy	Whole tumor(h)
227		1	3
238		1	3
237		2	3
231		2	3
225		3	3
236		3	3
228		1	6
230		6	6
552			24

Seven mice with large A2780 tumors and nine mice with small A2780 tumors were included in the study. The table shows time points for collection of biopsies and removal of whole tumors. Pretreatment biopsies were collected only from mice with large tumors.

### Experiment 4: Relationship between H4 acetylation in solid tumor and belinostat in plasma, tumor and spleen tissue

Groups of 3–4 mice with subcutaneous A2780 xenografts were treated with 200 mg/kg×1 belinostat i.v. or vehicle control (L-arginine 400 mg/kg in isotonic sterile saline) at time zero. After 15 min to 3 h, mice were sacrificed and plasma (lithium heparin stabilized), tumor, and spleen tissue were collected. Spleen and tumor were divided into two and prepared for analysis for belinostat content and H4 acetylation by IHC analysis. The degree of H4 acetylation was correlated with belinostat in plasma and tumor tissue. The spleen representing peripheral blood mononuclear cells (PBMCs) was included as reference tissue.

## RESULTS

### Reactivity of T25 anti-acetylated H4 antibody

**Fig. 2:**
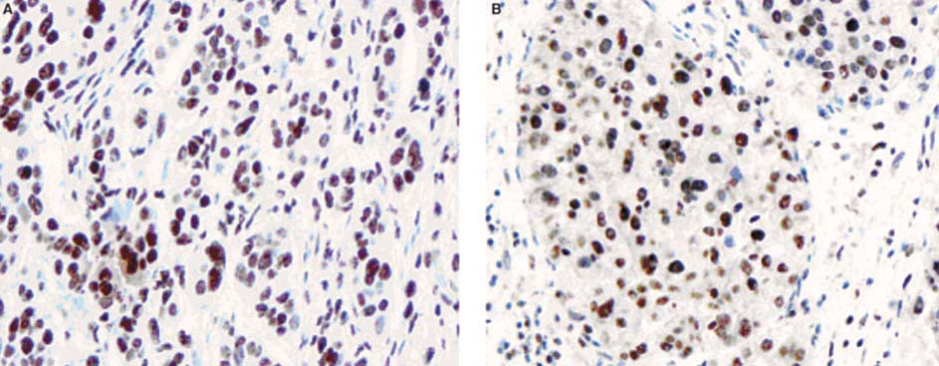
Nuclear expression of acetylated H4 in (A) breast and (B) liver carcinoma cells.

The antibody T25 performed well in immunohistochemistry on formalin-fixed paraffin-embedded tissue samples with no background staining; it showed nuclear labeling of epithelial and cancer cells in all tested specimens. Stromal cells showed no H4 acetylation. Acetylation of H4 in cancer tissues is shown in Fig. 2.

### Belinostat effect in different xenograft models (Experiment 1)

**Fig. 3:**
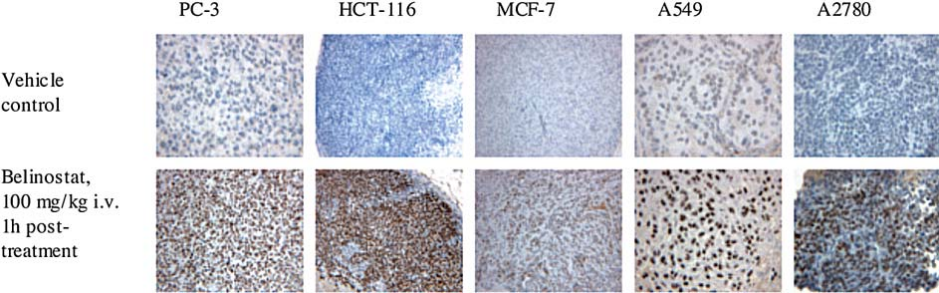
H4 acetylation *in vivo* in different subcutaneous xenograft models 1 h after treatment with belinostat (100 mg/kg i.v.) (Experiment 1).

The effect of belinostat on H4 acetylation was tested in the PC-3, HCT-116, MCF-7, A549 and A2780 model. A single i.v. injection of 100 mg/kg caused H4 acetylation after 1 h in all models. The strongest signal for H4 acetylation was found in the A2780 ovarian cancer model, followed by the HCT-116 colon cancer model and the A548 lung cancer model. Weak acetylation was seen in the PC-3 prostate cancer and the MCF-7 lung cancer model (Fig. 3).

### Collection of tumor biopsies and influence on H4 acetylation (Experiment 2)

Biopsies and corresponding solid tumor (500–1000 mg) from A2780 (15 mice), HCT-116 (12 mice), PC-3 (6 mice), and MCF-7 (4 mice) were compared to ensure that the biopsies taken were representative for the whole tumor, and that the models were suitable for biopsy collection.

All 15 biopsies collected from A2780 xenograft mice contained tumor tissue with preserved or partly preserved morphology, and only minimal necrosis. All 12 HCT-116 tumors contained liquid necrosis in their center, and viable tumor tissue was only observed in the periphery of the tumor. Consequently, it was only possible to collect a tumor tissue sample from 1 of 12 biopsies taken. All six PC-3 tumors contained large necrotic areas and only one biopsy contained tumor cells. Two of four biopsies from MCF-7 tumors contained tumor tissue. However, one of these biopsies contained necrosis as well.

**Fig. 4:**
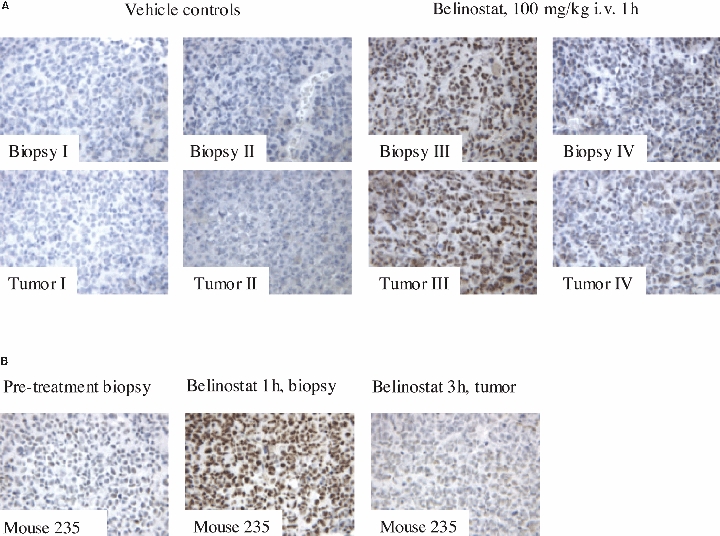
Acetylation of H4 in A2780 xenograft mice before and after belinostat treatment. (A) Acetylated H4 in four biopsies and their representative tumors (I–IV) (Experiment 2). No (A) or weak (IZI) H4 acetylation is observed in vehicle control mice, whereas strong (III) or moderate (IV) H4 acetylation is observed 1 h after intravenous treatment with belinostat (100 mg/kg) in both biopsies and tumors. (B) H4 acetylation *in vivo* in biopsies and tumor from the same mouse during belinostat treatment (100 mg/kg i.v.) (Experiment 3). The pretreatment biopsy shows weak H4 acetylation, whereas strong H4 acetylation is seen 1 h after treatment. After 3 h, the H4 acetylation is weak and has decreased to a level similar to that in the pretreatment biopsy.

H4 acetylation was compared between the biopsies and the representative tumors to ensure that biopsy collection itself did not have an impact on H4 acetylation. Of the 15 A2780 biopsies, 7 were treated with belinostat and the staining profile of acetylated H4 was similar in tumors and representative biopsies. In contrast, no or only weak H4 acetylation was observed in A2780 tumors and biopsies from eight vehicle-treated control mice. Examples of the staining are found in Fig. 4A. It was also investigated whether the repeated biopsy sampling had any effect on acetylation in A2780 tumors. Even though the repeat biopsies contained blood, this did not interfere with the IHC staining or interpretation of the results.

### Time dependence of belinostat treatment on H4 acetylation in solid tumor (Experiment 3)

The A2780 xenograft model was selected for monitoring time dependence of belinostat effect *in vivo*. 16 mice with either small (300–400 mg) or large (1500–1800 mg) A2780 tumors were treated with 100 mg/kg belinostat i.v. at time zero. Pretreatment (only large tumors) and post-treatment biopsies were collected from mice after 1, 2, 3, or 6 h. At sacrifice the whole tumor was excised.

**Fig. 5:**
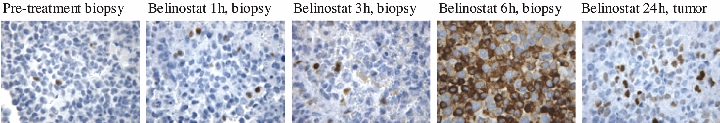
Expression of p21 in A2780 xenograft mice before and after belinostat treatment. p21 is observed in the nucleus of occasional cells in samples collected pretreatment, and up to 3 h after treatment. Weak cytoplasmic expression of p21 is observed 3 h after treatment and is strongly increased 6 h after treatment. The cytoplasmic expression of p21 disappears after 24 h. However, the nucleic expression is now more pronounced.

22 biopsies from 16 mice were collected during the study, and 21 of these biopsies contained tumor tissue. Results of H4 acetylation are shown in Table 2. Examples of staining are shown in Fig. 4B. Pretreatment biopsies (n=7) showed no (n=1), weak (n=3) or moderate (n=2) H4 acetylation. One pretreatment biopsy did not contain tumor tissue. All biopsies collected 1 h (n=5) and 2 h (n=4) after belinostat treatment showed strong H4 acetylation; 3 h after treatment strong acetylation was observed in 1/10 removed tumors as well as in 2 biopsies from 3 of these tumors. The general findings at 3 h were weak or moderate acetylation similar to the levels observed in the pretreatment biopsies. After 6 and 24 h, weak or no acetylation was observed. Results of the p21 expression are shown in Fig. 5. p21 showed strong nucleic expression in occasional cells from biopsies collected pretreatment, and 1 and 2 h after treatment. In addition, weak cytoplasmic expression was observed in two of six pretreatment biopsies (33%), while one of four biopsies collected after 2 h showed moderate cytoplasmic expression (25%). The cytoplasmic expression of p21 was increased after 3 and 6 h, respectively. Thus, of 10 tumors removed after 3 h, 5 (50%) showed weak expression, 2 (20%) showed moderate expression, and one (10%) showed strong expression. After 6 h, three of four tumors (75%) showed moderate expression and one of four (25%) showed strong expression. All biopsies removed after 6 h showed moderate-to-strong cytoplasmic expression as well. After 24 h, the cytoplasmic expression had disappeared. In contrast, nucleic staining was more pronounced than in the earlier samples.

**TABLE 2:** *Results of H4 acetylation in A2780 biopsies and tumors during belinostat treatment* in vivo *(Experiment 3)*

A2780	*H4 acetylation*							
		Belinostat, 100 mg/kg i.v.						
ID	Pretreatment biopsy	1 hbiopsy	2 hbiopsy	3 hbiopsy	3 htumor	6 hbiopsy	6 htumor	24 htumor
Large tumors (1500–1800 mg)								
235	Weak	Strong			Weak			
234	Moderate	Strong			Moderate			
226	Weak		Strong		Weak			
365	Moderate		Strong		Moderate			
232	Weak			Strong	Moderate			
233	No					No	No	
553	–					Weak	Weak	
Small tumors (300–400 mg)								
227		Strong			Weak			
238		Strong			Moderate			
237			Strong		Strong			
231			Strong		–			
225				Moderate	Moderate			
236				Strong	Moderate			
228		Strong					Weak	
230						Weak	Weak	
552								No

Strong H4 acetylation is observed in biopsies collected 1 and 2 h after treatment. In most samples, acetylation is decreased after 3 h to a level similar to that observed in the pretreatment biopsies. Only weak acetylation is observed 6 h after treatment. (–, missing).

### Relationship between H4 acetylation in solid tumor and belinostat levels in plasma, tumor and spleen tissue (Experiment 4)

Mice with subcutaneous A2780 xenografts (500–1000 mg) were treated with belinostat (200 mg/kg i.v.) or vehicle control and sacrificed after 15 min, 30 min, 1 h, 2 h, or 3 h for collection of plasma, tumor and spleen for analysis of belinostat content and staining for acetylated H4 in tissue. The belinostat dose was increased from 100 to 200 mg/kg to increase the possibility of detection of belinostat in tumor and spleen tissue and to investigate whether the acetylation period could be extended beyond the 1–2 h period observed in Experiment 3.

**Fig. 6:**
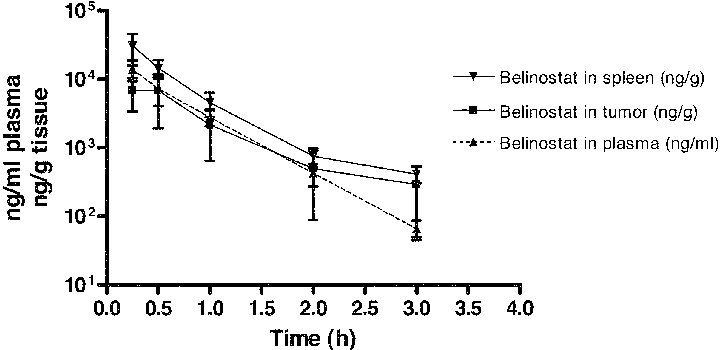
Belinostat in plasma (ng/ml), A2780 subcutaneous tumor (ng/g) and spleen (ng/g) after intravenous treatment with 200 mg/kg single dose at 0 min (Experiment 4). The distribution of belinostat to tumor tissue follows the plasma pharmacokinetic curve the first 2 h after treatment. After 3 h, the concentration of belinostat in plasma seems to decline more than that in the tumor and spleen tissue. The distribution of belinostat to subcutaneous solid tumor is comparable to the spleen distribution.

Analysis of belinostat in plasma, tumor and spleen showed that its distribution to tumor and spleen tissue was similar, and that tissue and plasma distribution curves were parallel (Fig. 6). Thus, belinostat plasma concentration seems to reflect the actual belinostat concentrations in subcutaneous solid tumor tissue.

**Fig. 7:**
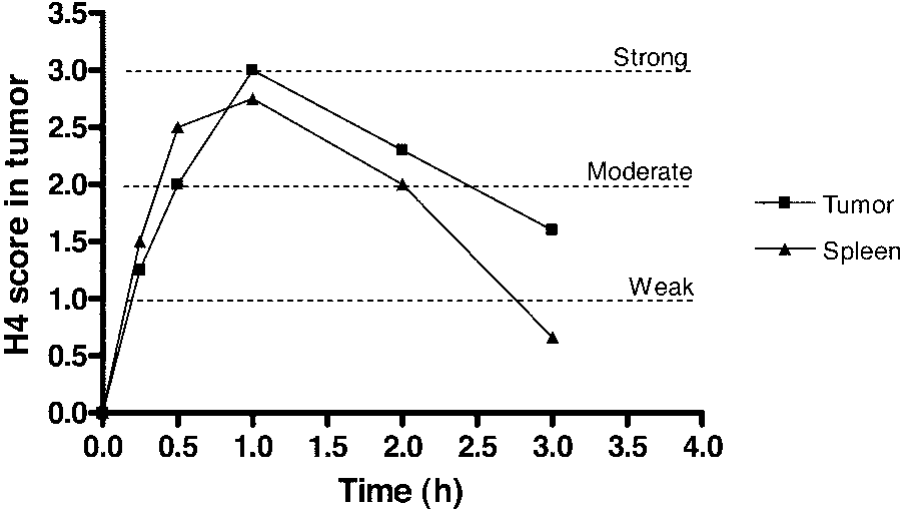
H4 acetylation score in tumor and spleen tissue after treatment with belinostat, 200 mg/kg i.v. Maximal acetylation is observed 1 h after treatment. The acetylation in spleen and subcutaneous tumor is comparable. A2780 xenograft, NMRI nude mice, 3–4 mice/time point.

**Fig. 8:**
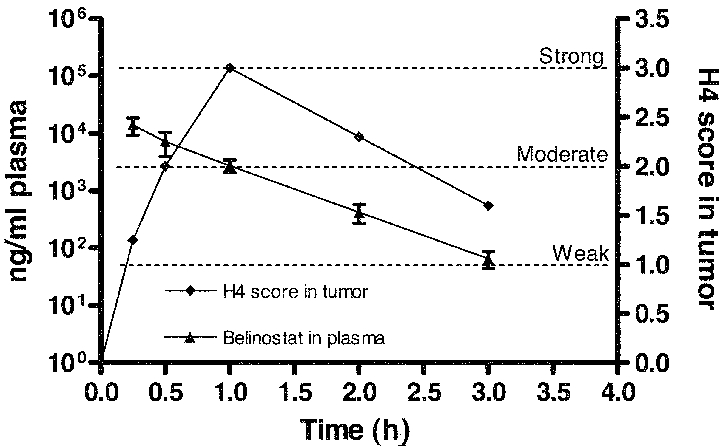
Overlay plot of belinostat concentrations in plasma and H4 acetylation score in tumor tissue after treatment with belinostat, 200 mg/kg i.v. Maximal acetylation is observed 1 h after treatment, which coincides with belinostat plasma concentrations above 10^3^ ng/ml. A2780 xenograft, NMRI nude mice, 3–4 mice/time point.

Results of H4 acetylation are shown in Table 3. After 15 min, weak-to-moderate acetylation was observed in three of four tumors. Maximal H4 acetylation levels were reached at 1 h, with four of four tumors showing strong H4 acetylation. After 2 h, one of three tumors showed strong acetylation, but after 3 h, the acetylation had decreased to weak and moderate levels. Similar levels of H4 acetylation were observed for spleen tissue (Fig. 7). No H4 acetylation was detected in spleen and tumor tissue from vehicle control mice. When correlating the plasma pharmacokinetic profile with H4 acetylation in solid tumor tissue, the present study found that belinostat activity, as indicated by the acetylated H4 surrogate marker, was present as long as the plasma concentration was above the 1000 ng/ml level (Fig. 8). As belinostat plasma concentrations decrease, the H4 acetylation in tumor tissue also declines.

**TABLE 3:** Results of H4 acetylation (score) in tumors and spleens from vehicle control mice or belinostat-treated mice with A2780 xenografts (Experiment 4)

A2780	H4 acetylation					
		Belinostat, 200 mg/kg i.v.				
ID	Vehicle, 15 min	15 min	30 min	1 h	2 h	3 h
Tumor						
270	No (0)					
478	No (0)					
282	No (0)					
524		Weak (1)				
277		Moderate (2)				
531		Moderate (2)				
296		No (0)				
273			Moderate (2)			
286			Moderate (2)			
523			Strong (3)			
527			Weak (1)			
511				Strong (3)		
283				Strong (3)		
525				Strong (3)		
508				Strong (3)		
287					Moderate (2)	
285					Strong (3)	
522					Moderate (2)	
532						Moderate (2)
295						Weak (1)
279						Moderate (2)
Mean	0	1.25	2	3	2.33	1.67
Spleen						
270	No (0)					
478	No (0)					
282	No (0)					
524		Moderate (2)				
277		Moderate (2)				
531		Weak (1)				
296		Weak (1)				
273			Strong (3)			
286			Strong (3)			
523			Moderate (2)			
527			Moderate (2)			
511				Strong (3)		
283				Strong (3)		
525				Moderate (2)		
508				Strong (3)		
287					Moderate (2)	
285					Moderate (2)	
522					Moderate (2)	
532						Weak (1)
295						No (0)
279						Weak (1)
Mean	0	1.5	2.5	2.75	2	0.67

No H4 acetylation is observed in tissue from vehicle control mice. Weak-to-moderate acetylation is observed after 15 min with maximal levels after 1 h. The levels of acetylation decrease after 2 h.

## DISCUSSION

Histone acetylation in PBMCs is often used as a surrogate marker for HDAC inhibitor activity and has shown a correlation with plasma concentration (19). Pharmacokinetic studies have shown linear pharmacokinetics with dose proportional increases in C_max_ and AUC for vorinostat (12) and linear, dose-independent pharmacokinetics for MS-275 (18). Although time to C_max_ has shown similarity between different HDAC inhibitors (C_max_ reached after 1–2 h), there have been large variations in the terminal half-life between HDAC inhibitors (ranging from 21 min to 74 h) (12, 14, 18, 23).

Evaluation of five cell lines grown as xenografts on nude mice revealed differences in their utility as biopsy models (Experiments 1 and 2), which was probably due to differences in the necrotic tendency of the tumors. PC-3 and MCF-7 tumors showed less acetylation after 1 h compared to A2780 tumors. It is unknown whether this difference in acetylation is due to decreased efficacy of the drug in certain tissues or due to differences in cell viability. The PC-3, MCF-7, and HCT-116 model contained necrotic and fluid areas, which hindered biopsy collection. Biopsies from A2780 tumors contained vital tumor tissue, making this a more suitable model. In addition, A2780 biopsies were representative of the tumors regarding level of H4 acetylation. Our initial studies showed that the method of biopsy collection had no influence on histone acetylation.

H4 acetylation was detected in tumor tissue 15 min after belinostat treatment and the maximum level was reached after 1 h. In a previous study, increased H4 acetylation was found in peripheral canine blood 10 min after i.v. infusion of belinostat given over 45 min (total dose 50 mg/kg) (24). In addition, the investigators found increased H4 acetylation 30 min after treatment with 1 μM belinostat in histones extracted from A2780 cells (24). We observed that two biopsies collected after 3 h showed stronger acetylation than the corresponding tumors. Staining of the tumors was uneven with weaker acetylation in the middle than in the periphery. This problem might be a result of insufficient fixation or too short epitope retrieval of the tumors. Nevertheless, our results showed that H4 acetylation reached base line level 3 h after treatment. Similarly, Plumb et al. found that H4 acetylation had returned to base line levels in histones extracted from A2780 cells and peripheral blood from A2780 xenografts 3 h after treatment with belinostat (1 μM or 40 mg/kg i.p.) (24). Our results from the monitoring study (Experiment 3) showed different levels of H4 acetylation in pretreatment biopsies from different mice (Table 2), which indicates that it is important to collect pretreatment biopsies in order to have an internal control for baseline H4 acetylation.

In a clinical study, higher doses of the HDAC inhibitor Zolinza™ did not produce an increase in extent of histone acetylation in peripheral blood lymphocytes. Instead, the half-life of acetylated histones seemed to be increased (13). In our study, increasing the dose from 100 to 200 mg/kg did not result in an extended acetylation period beyond the 1–2 h observed in Experiment 3.

p21 was induced in the cytoplasm about 3 h after belinostat treatment, and was further increased after 6 h, where strong cytoplasmic expression was observed. Thus, p21 was induced after acetylation of H4 was observed. H4 acetylation thus correlates with activation of gene transcription after treatment with belinostat. The cytoplasmic expression of p21 was decreased after 24 h. In contrast, p21 had relocated to the nucleus. Cytoplasmic induction of p21 has previously been reported by O'Reilly et al. (25). Here, the cytoplasmic induction was observed in mice exposed to hyperoxia for 64 h.

We found comparable belinostat distribution to tumor and spleen tissue, and in addition that the distribution in tumor tissue followed the belinostat plasma pharmacokinetics. This indicates that belinostat distribution is not tumor specific and that plasma concentrations of belinostat may be used to predict belinostat distribution to solid tumor tissue. In the present study, the belinostat concentration in plasma and tumor correlated with levels of acetylated H4 in the solid tumor tissue and PBMCs. Acetylated H4 is a defining event after treatment with an HDAC inhibitor, and can thus be used as an indicator of belinostat activity. We found that H4 acetylation was present as long as the plasma concentration was above 1000 ng/ml. This supports that maintenance of belinostat plasma levels above a certain level is required in order to achieve a prolonged effect of belinostat treatment in solid tumors.

In conclusion, the monoclonal antibody T25 against acetylated H4 worked well on formalin-fixed paraffin-embedded tissue samples. The effect of belinostat on H4 acetylation using the T25 antibody was evident 1 and 2 h after dosing, with a return to baseline levels after 3 h. H4 acetylation followed the belinostat concentration in tumor tissue. Examination of H4 acetylation in fine needle biopsies using the T25 antibody may thus prove useful in monitoring HDAC inhibitor efficacy in clinical trials involving solid tumors.
